# Assessment of Serum Lipocalin-2 in Type 2 Diabetes Mellitus Patients With and Without Retinopathy

**DOI:** 10.7759/cureus.111231

**Published:** 2026-06-21

**Authors:** Sanaur Naz, Gandhipuram Periyasamy Senthilkumar, Sherin Elizabeth Thomas, Rajesh Panneerselvam Anandhi, Ramesh Babu K

**Affiliations:** 1 Department of Biochemistry, Jawaharlal Institute of Postgraduate Medical Education and Research (JIPMER), Puducherry, IND; 2 Department of Ophthalmology, Jawaharlal Institute of Postgraduate Medical Education and Research (JIPMER), Puducherry, IND

**Keywords:** diabetic retinopathy, inflammation, lipocalin-2, tumour necrosis factor-alpha (tnf-alpha), type 2 diabetes mellitus

## Abstract

Background: Diabetic retinopathy (DR) is a serious microvascular complication of diabetes mellitus. Recent studies have shown elevated lipocalin-2 in complications associated with type 2 diabetes mellitus (T2DM). These elevated lipocalin-2 levels have been linked with dysregulated lipid metabolism and inflammation, and they may potentially contribute to the disease severity of DR.

Objective: The study aimed to assess and compare serum levels of lipocalin-2 in T2DM patients with and without retinopathy and to uncover its role in the progression of DR.

Methods: This study involved 82 T2DM patients divided into two groups according to the presence (n=41) or absence of DR (n=41). Clinical parameters like glucose, lipid profile (triglyceride, very low-density lipoprotein (VLDL), low-density lipoprotein (LDL), high-density lipoprotein (HDL), and total cholesterol), urea, and creatinine values were analyzed using an autoanalyzer. The anthropometric parameters were measured, and an enzyme-linked immunosorbent assay (ELISA) for lipocalin-2 and tumor necrosis factor-alpha (TNF-alpha) was performed.

Results: Serum levels of lipocalin-2 (<0.01) and TNF-alpha (<0.01) were significantly elevated in patients with DR compared to those without DR.

Conclusion: The findings suggest that lipocalin-2 and TNF-alpha could serve as potential circulating biomarkers for DR in individuals with T2DM. Their significant association with DR emphasizes their possible role in early identification of risk and monitoring of disease progression.

## Introduction

Type 2 diabetes mellitus (T2DM), a common chronic disease, is currently affecting more than 400 million individuals worldwide as a result of both genetic and environmental causes. The pathogenesis of the disease consists of insulin resistance and impairment of β-cells, with processes such as β-cell dedifferentiation, mitochondrial impairment, and oxidative stress being involved and culminating in constantly high blood sugar levels. The chronic increase in blood glucose level causes microvascular damage to different tissues, especially the retina, where chronic metabolic and oxidative stress impairs the vascular integrity [1]. Consequently, diabetic retinopathy (DR) becomes one of the most prevalent microvascular issues of diabetes and a significant cause of vision loss among all age groups: children, adults, and the elderly. The existing therapies used to treat DR are intravitreal injections with anti-vascular endothelial growth factor therapies, vitreous surgery, and laser therapy. Nevertheless, not all diabetic patients with diabetic macular edema (DME) undergo meaningful changes in vision after two years of treatment; only 29% of diabetic patients do. The high cost and frequency of antibody-based therapies also pose a financial and practical burden to patients. Although keeping blood sugar levels under control can slow the development and progression of DR, some research shows that DR can still get worse in patients with strict glycemic management because of a process called “metabolic memory,” which is not yet well understood [2].

In order to develop earlier management strategies and prevent or delay the onset of vascular complications, several molecular markers are being studied. An important aspect in the pathophysiology of DR involves potential inflammatory and angiogenic proteins. Their intracellular role and systemic signaling functions could link the systemic changes to local changes occurring at the site of microvascular pathology.

Lipocalin-2 holds great significance in macrovascular complications of T2DM. It has emerged as an important adipokine and inflammatory mediator, with growing evidence highlighting its involvement in metabolic dysregulation and vascular pathology associated with diabetes. It has been associated with endothelial dysfunction, dysregulation of glucose homeostasis, and signaling associated with lipid metabolism and inflammatory pathways. Through these mechanisms, lipocalin-2 may contribute to oxidative stress, chronic low-grade inflammation, and vascular damage, all of which are central to the pathogenesis of diabetic complications. Based on the literature, its role in diabetic retinopathy may be linked to dysregulated lipid metabolism and inflammation, and it may potentially contribute to the disease severity of DR [3]. These processes are known to exacerbate retinal microvascular damage, increase vascular permeability, and promote neurodegeneration in the diabetic retina, thereby influencing both the onset and progression of diabetic retinopathy. Moreover, altered lipid handling and inflammatory signaling in retinal tissues may further amplify lipocalin-2-mediated pathogenic effects.

Moreover, there is considerable evidence on the role of lipocalin-2 in retinal degradation in age-related macular degeneration (AMD), whereas, in DR, the reports are so far quite scarce. AMD studies indicate that lipocalin-2 may play a role in retinal inflammation, extracellular matrix remodeling, and neuronal damage, which implies that lipocalin-2 may have a wider role in retinal degenerative diseases [4]. The limited data available in DR underscores a significant gap in current knowledge. Hence, investigating the levels of lipocalin-2 and understanding its association with DR is essential.

Lipocalin-2 is a unique adipokine that is associated with DR. It is well known to contribute to the pathology of macrovascular complications and renal disease in diabetes [3]. But, to our knowledge, only a limited number of studies have investigated lipocalin-2 in patients with type 2 DR. The novel objective explored in this study is to uncover the role of circulating lipocalin-2 levels in the progression of DR.

## Materials and methods

Ethical considerations

Prior to participant recruitment, approval for the study was obtained from the Departmental Postgraduate Research Monitoring Committee and the Institutional Ethics Committee of Jawaharlal Institute of Postgraduate Medical Education and Research (JIPMER), Puducherry, India (JIP/IEC-OS/2024/42; dated April 20, 2024). The study was conducted in accordance with the 2017 Indian Council of Medical Research (ICMR) Revised National Ethical Guidelines for Biomedical and Health Research involving Human Participants [5]. All participants received a detailed explanation regarding the study protocol, and written informed consent was obtained from every individual before enrollment.

Study setting and participant enrollment

This cross-sectional observational study was carried out among patients with T2DM aged between 35 and 65 years attending the ophthalmology outpatient department of a tertiary care hospital in Puducherry, India. The duration of the study was from April 2024 to March 2026, and the participants were recruited based on a convenience sampling technique. The presence of DR was confirmed via comprehensive fundoscopy reports. All fundoscopic evaluations were performed by a qualified ophthalmologist using the standardized International Clinical Diabetic Retinopathy Disease Severity Scale to establish a definitive diagnosis [6]. Based on these criteria, participants were categorized using a binary classification system into two primary study groups: T2DM patients without retinopathy (Group 1, n = 41) and T2DM patients with retinopathy (Group 2, n = 41). Patients with non-diabetic ocular disorders such as AMD, malignancy, heart failure, renal failure, chronic inflammatory diseases, including arthritis, and infectious diseases were excluded from the study. Individuals with morbid obesity of a body mass index cutoff ≥ 32.5 kg/m² were excluded [7].

Sample size calculation

In order to obtain an expected mean difference in serum lipocalin-2 and tumor necrosis factor-alpha (TNF-alpha) between our two study groups, the following assumptions were made: Power = 80%, confidence interval = 95%, and alpha error = 5%. The sample size calculation was done based on the primary outcome. The serum lipocalin-2 levels obtained from an earlier study were used for the sample size estimation [8]. The OpenEpi sample size calculator for comparing two means (SSMean) (Version 3; Dean, A. G., Sullivan, K. M., & Soe, M. M. (2013). OpenEpi: Open Source Epidemiologic Statistics for Public Health (Version 3.01). www.OpenEpi.com) was used for the calculation. In each group, the study size reached 41.

Data collection and study variables

Anthropometric measurements, clinical history, and blood pressure parameters were documented for all participants. The presence of microvascular complications, particularly diabetic retinopathy, was confirmed using standard fundoscopy reports. Fasting venous blood samples were collected, and serum was separated for biochemical analysis. Serum glucose, lipid profile, urea, and creatinine levels were measured using the Beckman Coulter AU680 and AU5800 automated biochemistry analyzers (Beckman Coulter, Inc., Brea, CA, USA). Serum lipocalin-2 and TNF-α concentrations were estimated using enzyme-linked immunosorbent assay (ELISA) kits (Human Lipocalin-2 ELISA Kit and Human TNF-α ELISA Kit; Elabscience Biotechnology Inc., Wuhan, Hubei, China) according to the manufacturer's instructions. The Human Lipocalin-2 ELISA kit demonstrated an analytical sensitivity of 18.75 pg/mL, a detection range of 31.25-2000 pg/mL, and a repeatability coefficient of variation (CV) of <10%. The Human TNF-alpha ELISA kit demonstrated an analytical sensitivity of 4.69 pg/mL, a detection range of 7.81-500 pg/mL, and a repeatability CV of <10%. Aliquots intended for ELISA analysis were stored at −40°C for a maximum duration of six to eight months until the completion of data collection. To preserve protein integrity, all samples were thawed only once immediately prior to the execution of the assays, and repeated freeze-thaw cycles were strictly avoided.

Calculation of lipid indices

The triglyceride glucose index (TyG-Index) was calculated as $\mathrm{TyG-Index} = \ln \left( \frac{\text{fasting triglycerides } [\mathrm{mg/dL}] \times \text{fasting plasma glucose } [\mathrm{mg/dL}]}{2} \right)$ [9].

The triglyceride glucose-body mass index was calculated as $\mathrm{TyG-BMI} = \ln \left( \frac{\text{fasting glucose } [\mathrm{mg/dL}] \times \text{triglycerides } [\mathrm{mg/dL}]}{2} \right) \times \mathrm{BMI}$ [9].

The atherogenic index of plasma (AIP) was calculated as $\mathrm{AIP} = \log \left( \frac{\mathrm{TG}}{\mathrm{HDL-C}} \right)$ [10].

The atherogenic coefficient (AC) was calculated as $\mathrm{AC} = \frac{\mathrm{Non-HDL-C}}{\mathrm{HDL-C}}$ [10].

The Castelli risk index-I (CRI-I) was calculated as $\mathrm{CRI-I} = \frac{\mathrm{TC}}{\mathrm{HDL-C}}$ [10].

The Castelli risk index-II (CRI-II) was calculated as $\mathrm{CRI-II} = \frac{\mathrm{LDL-C}}{\mathrm{HDL-C}}$ [10].

Statistical analysis

**Figure 1:**
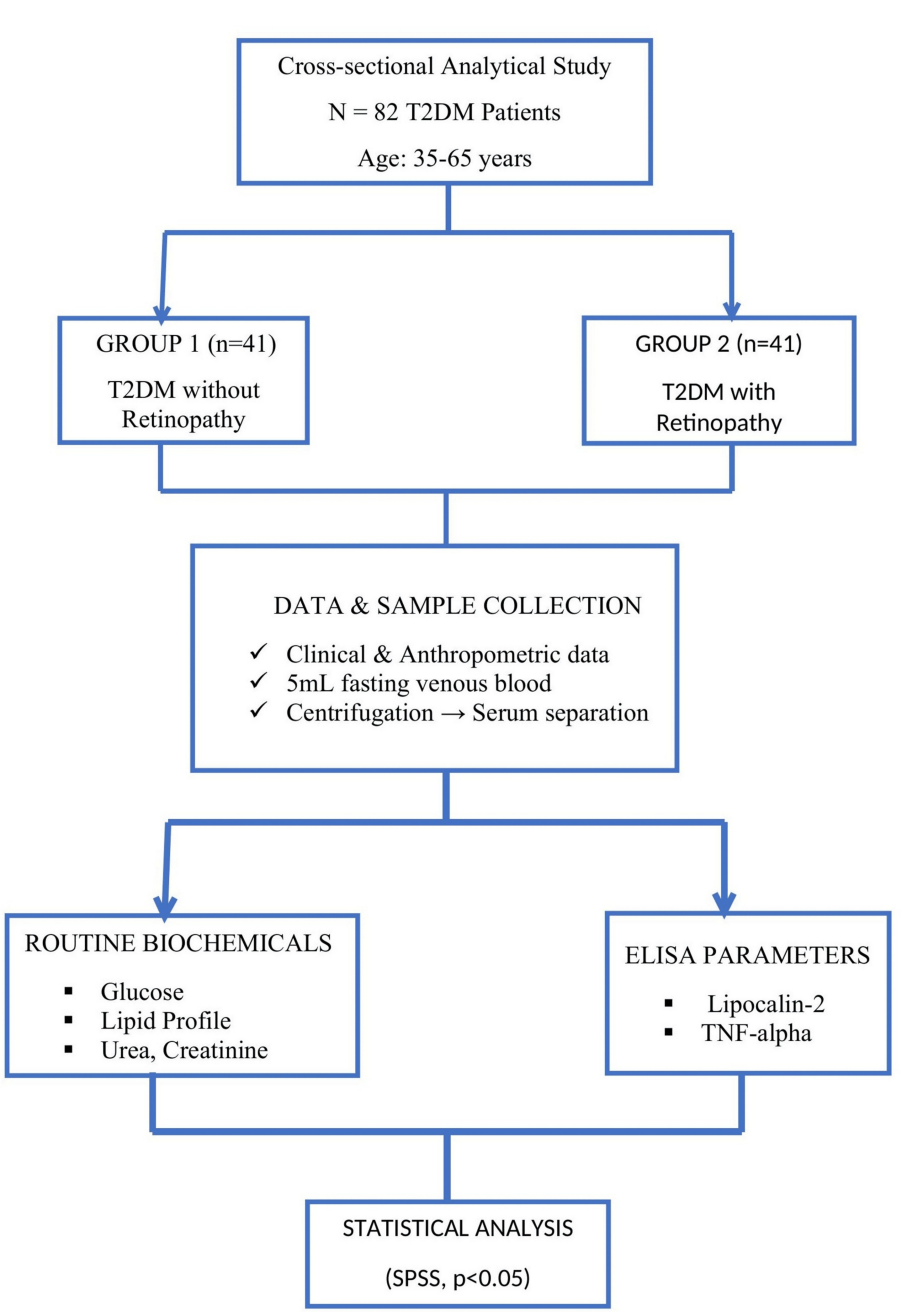
Study flow diagram Study flow diagram showing the design of the cross-sectional observational study conducted among 82 patients with T2DM. T2DM: type 2 diabetes mellitus; ELISA: enzyme-linked immunosorbent assay; TNF-alpha: tumor necrosis factor-alpha

All statistical analyses were performed using IBM SPSS Statistics software version 20.0 (IBM Corporation, Armonk, NY, USA). Normality of continuous variables was assessed using the Shapiro-Wilk test. Depending on the distribution, data were expressed as mean ± standard deviation (SD) or median with interquartile range (IQR). Categorical variables were presented as frequencies and percentages. Comparisons between groups were performed using the independent t-test or Mann-Whitney U test, as appropriate. A p-value < 0.05 was considered statistically significant for all analyses. The overall study methodology is illustrated in the study flow diagram (Figure 1).

## Results

In this cross‑sectional study, a total of 82 participants were included and were equally divided into two groups: T2DM without retinopathy (Group 1, n = 41) and T2DM with retinopathy (Group 2, n = 41).

We observed that the duration of diabetes was significantly greater in T2DM patients with retinopathy compared to those without retinopathy. But other anthropometric parameters did not show a significant increase in patients with DR than without DR. The comparison of anthropometric parameters between the study groups is presented in Table 1.

**Table 1:** Comparison of anthropometric parameters between study groups *P < 0.05 was considered statistically significant; ^a^Test statistic (t value from the independent Student's t-test); ^b^Test statistic (U value from the Mann–Whitney U test); ^c^Test statistic (χ² value from the chi-square test). BMI: body mass index; SBP: systolic blood pressure; DBP: diastolic blood pressure; T2DM: type 2 diabetes mellitus; HTN: hypertension; DM: diabetes mellitus

Parameters	T2DM without retinopathy (n=41)	T2DM with retinopathy (n=41)	P-value	Test statistic value
Age (years)	51.00(45.0-60.0)	56.00(47.0-61.0)	0.053	632.5^b^
Gender (Male/Female)	21(51.2%)/20(48.8%)	13(31.7%)/28(68.3%)	0.115	2.485^c^
Weight (kg)	66.46±9.182	65.29±12.17	0.624	0.492^a^
Height (cm)	158(155-163)	158(155-168)	0.625	788^b^
BMI (kg/m^2^)	25.10(23.6-28.0)	24.00(21.35-27.35)	0.120	673^b^
Hip circumference (cm)	101.22±8.864	98.24±11.13	0.184	1.339^a^
Waist circumference (cm)	91.78±8.475	90.15±12.91	0.50	0.677^a^
SBP (mmHg)	136.68±24.93	131.20±21.54	0.289	1.066^a^
DBP (mmHg)	78(70.0-90.0)	79.3(70.0-86.0)	0.826	8.7^b^
HTN duration (years)	0(0.0–9.5)	1.0(0.0–6.0)	0.97	837^b^
DM duration (years)	7.00 (2.0-10.0)	12.00 (6.0-16.0)	<0.01*	457^b^

Table 2 shows the comparison of routine biochemical parameters between the study groups. Among the routine biochemical parameters investigated, we found that fasting glucose, total cholesterol, triglycerides, low-density lipoprotein (LDL), very-low-density lipoprotein (VLDL), and urea levels were significantly higher in patients with DR compared to patients without DR, whereas high-density lipoprotein (HDL) and creatinine levels did not show a significant increase in patients with DR compared to those without DR.

**Table 2:** Comparison of routine biochemical parameters between study groups *P < 0.05 was considered statistically significant; ^a^Test statistic (t value from the independent Student's t-test); ^b^Test statistic (U value from the Mann–Whitney U test). HDL-C: high-density lipoprotein cholesterol; LDL-C: low-density lipoprotein cholesterol; VLDL-C: very low-density lipoprotein cholesterol; TG: triglycerides; T2DM: type 2 diabetes mellitus.

Parameters	T2DM without retinopathy (n=41)	T2DM with retinopathy (n=41)	P-value	Test statistic value
Fasting glucose (mg/dL)	142.00(97.5-219.0)	206.00(145.5-292)	0.012*	569.5^b^
Total cholesterol (mg/dL)	151.00(122-182)	196.00(169-240)	<0.01*	350^b^
Triglycerides (mg/dL)	134.00(98.0-206.5)	218.00(142.0-308.5)	<0.01*	557.5^b^
HDL-C (mg/dL)	38.00(33.1-45.9)	40.0(36.25-47.5)	0.132	678^b^
LDL-C (mg/dL)	93.44±29.365	131.05±34.79	<0.01*	-5.290^a^
VLDL-C (mg/dL)	27.00(19.5-41.5)	44.00(28.5-61.5)	<0.01*	553^b^
Urea (mg/dL)	22.00(18.5-30.0)	29.00(21.5-47.0)	0.013*	573^b^
Creatinine(mg/dL)	1.00(1.00-1.00)	0.90(0.80-1.65)	0.460	766.5^b^

Table 3 shows the comparison of calculated indices between study groups. The markers of dyslipidaemia and atherogenic risk were calculated based on routine biochemical investigations. Only the TyG-Index was significantly higher in T2DM with retinopathy compared to patients without DR.

**Table 3:** Comparison of calculated indices between study groups *P < 0.05 was considered statistically significant; ^a^Test statistic (t value from the independent Student's t-test); ^b^Test statistic (U value from the Mann–Whitney U test). TyG: triglyceride-glucose index; TyG-BMI: triglyceride-glucose body mass index; AIP: atherogenic index of plasma; AC: atherogenic coefficient; CRI: Castelli risk index; T2DM: type 2 diabetes mellitus.

Parameters	T2DM without retinopathy (n=41)	T2DM with retinopathy (n=41)	P-value	Test statistic value
TyG-BMI	129.19±17.16	129.24±27.32	0.99	-0.01^a^
TyG-Index	4.9(4.7-5.25)	5.20 (4.90–5.50)	0.017*	584.5^b^
AIP	0.588±0.27	0.610±0.29	0.727	-0.35^a^
AC	2.8(2.05-3.70)	3.60 (2.6-4.3)	0.088	656.5^b^
CRI-I	4(3.15-6.05)	4.60 (3.6-5.3)	0.55	776^b^
CRI-II	2.4(1.8-3.0)	2.90 (2.05-3.5)	0.106	666.5^b^

According to the objectives, we quantified serum levels of lipocalin-2 and TNF-alpha in the two study groups. Table 4 shows the comparison of these special parameters between study groups. Serum lipocalin-2 and TNF-alpha were significantly elevated in T2DM patients with retinopathy than without retinopathy.

**Table 4:** Comparison of lipocalin-2 and TNF-alpha between T2DM patients with and without retinopathy *P < 0.05 was considered statistically significant; ^b^Test statistic (U value from the Mann–Whitney U test). T2DM: type 2 diabetes mellitus; TNF-alpha: tumour necrosis factor-alpha

Parameters	T2DM without retinopathy (n=41)	T2DM with retinopathy (n=41)	p-value	Test statistic value
Lipocalin-2 (pg/mL)	316.0(268.5-391)	577.00(476.5-675.5)	<0.01*	89^b^
TNF-alpha (pg/mL)	8.00(6-12)	21.00 (17-29)	<0.01*	87.5^b^

## Discussion

This study was conducted to compare serum lipocalin-2 and serum TNF-alpha levels in T2DM with and without DR. We observed that lipocalin-2 levels were significantly greater in DR patients than in non-DR individuals.

Our findings are in concordance with previous studies that have reported elevated serum lipocalin-2 in patients with diabetic retinopathy. In a study by Chung et al., plasma lipocalin-2 levels positively correlated with DR and its severity in patients with T2DM [11]. Also, Huang et al. suggested that lipocalin-2 regulates glucose metabolism by partially blocking the inhibitory effects of insulin on glucose production and glucose-6-phosphatase expression in hepatocytes, thereby affecting glucose absorption [12]. They further proposed that lipocalin-2 may contribute to chronic low-level systemic inflammation and insulin resistance. Studies on microvascular degeneration, like Ong et al., have demonstrated that lipocalin-2, which may be vasotoxic, uses iron chelation to mediate innate immune responses to starve bacteria [13]. Additionally, lipocalin-2 can also carry iron to the cytoplasm [14], where iron can produce potent reactive oxygen species that will induce oxidative stress and contribute to the rapid loss of endothelial cells in retinal microvessels and increased vascular permeability [15]. These are the two possible mechanisms as to why lipocalin-2 might cause DR, but the exact mechanism is yet unknown. It has been observed that, in addition to inflammation, insulin resistance and microvascular degeneration play an important role in the pathogenesis of DR as independent pathways [16].

Similarly, TNF-alpha, an inflammatory marker, was significantly elevated in DR patients compared to patients without DR. This observation aligns with its known role in chronic inflammation and in the development of microvascular complications in diabetes. Elevated TNF-alpha may contribute to retinal and endothelial cell injury in diabetes by inducing apoptosis directly via death receptors and indirectly through leukostasis, which might lead to vascular leakage and retinal damage [17]. A study by Costagliola et al. reported that TNF-alpha levels are linked with DR after adjusting for potential confounders and suggested that the level of TNF-alpha may be connected with clinical disease severity as well as with the predictors of microvascular damage, BMI, and glycated hemoglobin (HbA1c) [18]. Multiple studies further demonstrate that circulating and soluble TNF receptor levels correlate with the severity and progression of DR, particularly in proliferative stages [19].

In accordance with previous studies, our findings suggest that both lipocalin-2 and TNF-alpha may act as important inflammatory mediators in DR, and their combined action may exacerbate vascular complications. Our assumption that both lipocalin-2 and TNF-alpha play an important role in the pathogenesis of DR was supported by the findings of Law et al., which demonstrated that lipocalin-2 can activate the arachidonate 12-lipoxygenase metabolic pathway and promote adipose expression of TNF-alpha, which can increase local inflammation, disrupt energy balance, and result in systemic insulin resistance [20,21].

In our study, we observed a significant increase in several routine parameters and atherogenic markers, namely the fasting glucose, total cholesterol, triglycerides, LDL, VLDL, urea, and TyG-Index in DR patients, similar to previous studies. This finding suggests a progressive deterioration in metabolic and lipid homeostasis with increasing severity of DR [11]. We also found that patients with DR had a significantly longer duration of diabetes than those without retinopathy (12 vs. seven years, p<0.01). As diabetes duration is an established risk factor for DR, it may have contributed to the observed group differences. However, lipocalin-2 and TNF-alpha levels remained significantly elevated in the retinopathy group, supporting their potential association with the disease. Further studies with larger, duration-matched cohorts are needed to evaluate their independent role in DR.

Interestingly, our study showed that BMI and hip and waist circumference were lower in the DR patients compared to the patients without retinopathy. Numerous epidemiological studies have investigated the association of DR with BMI or anthropometric factors; the results have been conflicting. For instance, Hwang et al. conducted a study on the Korean population showing an inverse relation of BMI and waist circumference with the presence and severity of DR [22]. However, studies from Western nations have shown contradictory findings. The Hoorn study in the Netherlands reported a significant effect of higher BMI on DR. This discrepancy in results might be explained partly by variations in study methodology as well as by ethnic differences in study participants [23]. The observed alterations in biochemical and anthropometric parameters suggest that the progression of retinopathy is multifactorial, with inflammation and dyslipidemia playing significant contributory roles.

One of the main advantages of this study lies in its comprehensive evaluation of both conventional clinical parameters and emerging biochemical markers for DR. By assessing the duration of diabetes, lipid profile, lipocalin-2, and TNF-alpha, the study integrates markers of glycemic control, metabolic dysfunction, and inflammation, thereby providing a well-rounded risk assessment. Importantly, the study adds to the limited literature on lipocalin-2's role with TNF-alpha in DR and presents it as a marker for early risk assessment.

Despite its strengths, this study has some limitations that should be considered. The study population included only individuals with T2DM, without comparison to healthy, non-diabetic controls. As a result, the findings reflect biomarker variations within the diabetic population but do not establish whether these levels differ from those in healthy individuals. Furthermore, the role of lipocalin-2 within the diabetic subgroups (non-proliferative DR and proliferative DR) would have provided a better picture of the role of lipocalin-2 in the progression of DR.

## Conclusions

This study highlights the potential roles of circulating biomarkers, lipocalin-2 and TNF-alpha, in the pathophysiology and progression of DR among patients with T2DM. The significant elevations in the routine lipid profile (triglycerides and LDL-C) and the novel inflammatory markers TNF-alpha and lipocalin-2 in the DR group underscore that DR is not solely a disease of the retinal microvasculature but a manifestation of widespread systemic, metabolic, and inflammatory dysregulation. These findings suggest that a comprehensive management strategy for T2DM, aimed not only at stringent glucose control but also at correcting dyslipidemia and modulating the inflammatory response, may help slow the progression of DR. Our results highlight the importance of early identification of high-risk individuals through biomarker-based screening, which could ultimately aid in timely intervention and improved disease management. Further large-scale longitudinal studies are needed to validate the roles of lipocalin-2 and TNF-alpha in DR progression. Inclusion of healthy controls may help determine whether these biomarkers are specific to diabetes or DR pathogenesis.
